# Multiple Sclerosis Diagnosis Using Machine Learning and Deep Learning: Challenges and Opportunities

**DOI:** 10.3390/s22207856

**Published:** 2022-10-16

**Authors:** Nida Aslam, Irfan Ullah Khan, Asma Bashamakh, Fatima A. Alghool, Menna Aboulnour, Noorah M. Alsuwayan, Rawa’a K. Alturaif, Samiha Brahimi, Sumayh S. Aljameel, Kholoud Al Ghamdi

**Affiliations:** 1 Department of Computer Science, College of Computer Science and Information Technology, Imam Abdulrahman Bin Faisal University, P.O. Box 1982, Dammam 31441, Saudi Arabia; 2 Department of Computer Information Systems, College of Computer Science and Information Technology, Imam Abdulrahman Bin Faisal University, P.O. Box 1982, Dammam 31441, Saudi Arabia; 3 Department of Physiology, College of Medicine, Imam Abdulrahman Bin Faisal University, P.O. Box 1982, Dammam 31441, Saudi Arabia

**Keywords:** artificial intelligence, multiple sclerosis, machine learning, deep learning, diagnosis, magnetic resonance imaging (MRI), clinical data

## Abstract

Multiple Sclerosis (MS) is a disease that impacts the central nervous system (CNS), which can lead to brain, spinal cord, and optic nerve problems. A total of 2.8 million are estimated to suffer from MS. Globally, a new case of MS is reported every five minutes. In this review, we discuss the proposed approaches to diagnosing MS using machine learning (ML) published between 2011 and 2022. Numerous models have been developed using different types of data, including magnetic resonance imaging (MRI) and clinical data. We identified the methods that achieved the best results in diagnosing MS. The most implemented approaches are SVM, RF, and CNN. Moreover, we discussed the challenges and opportunities in MS diagnosis to improve AI systems to enable researchers and practitioners to enhance their approaches and improve the automated diagnosis of MS. The challenges faced by automated MS diagnosis include difficulty distinguishing the disease from other diseases showing similar symptoms, protecting the confidentiality of the patients’ data, achieving reliable ML models that are also easily understood by non-experts, and the difficulty of collecting a large reliable dataset. Moreover, we discussed several opportunities in the field such as the implementation of secure platforms, employing better AI solutions, developing better disease prognosis systems, combining more than one data type for better MS prediction and using OCT data for diagnosis, utilizing larger, multi-center datasets to improve the reliability of the developed models, and commercialization.

## 1. Introduction

Multiple sclerosis (MS) is an autoimmune chronic demyelinating disease that impacts the central nervous system (CNS). It is characterized mainly by inflammation and neurodegeneration. Pathologically, the disease is manifested by MS plaques or lesions. These are focal areas of demyelination affecting predominantly the white matter of the central nervous system. MS has four types which are relapsing-remitting MS (RRMS), primary-progressive MS (PPMS), secondary-progressive MS (SPMS), and progressive-relapsing MS (PRMS) [1].

A total of 2.8 million are estimated to suffer from MS globally, with a prevalence rate of 35.9 per 100,000 [2]. Globally, a new case of MS is reported every five minutes [3]. MS mainly occurs in young adults, and is more common among females [4]. MS symptoms vary widely among patients. Symptoms include weak limbs, blurred vision, dizziness, fatigue, and tingling sensations [3].There is no definite cause for MS. However, research suggests that environmental factors play a role in triggering the disease in genetically susceptible individuals [5].

A reliable and precise diagnosis of MS is critical for enabling early interventions for the disease, as disease-modifying drugs aid in managing symptoms and preventing disease progression [6]. The diagnosis of MS is based on the presence of CNS lesions that are separated in both time and space and on the exclusion of all other diseases that mimic MS both clinically and radiologically [7]. There is no certain laboratory test for the diagnosis of the disease [8]. Therefore, the current 2017 McDonald diagnostic criteria for MS combine clinical assessment, imaging, and laboratory findings [9].

Magnetic resonance imaging (MRI) is currently the most effective tool for the diagnosis of MS [10], understanding the course of the disease, and examining the effects of treatments in experiments [11]. However, MS diagnosis using MRI is time-consuming, tiresome, and susceptible to manual errors. Therefore, artificial intelligence (AI) is being used to automate MS diagnosis using machine learning (ML) and deep learning (DL) techniques [12,13]. ML is a type of AI where computers are given the opportunity to learn without being explicitly programmed, while DL is a subset of ML composed of algorithms permitting the software to train itself to perform tasks by exposing multilayered neural networks to vast amounts of data.

Several papers have performed a review of the past research in MS diagnosis using AI techniques such as [12] that reviewed most previous papers that used DL techniques for the automated diagnosis of MS through MRI scans. They discussed the most used preprocessing techniques and presented the current challenges and possible future research opportunities.

In addition, Arani et al. [14] aimed to find the most efficient methods and techniques used for MS diagnosis. The authors analyzed the performance of those methods to recommend the most adequate one. They found that rule-based, fuzzy logic (FL), and artificial neural network (ANN) are the most widely used methods for diagnosing MS. They also identified the limitations of all these techniques and recommended using a combination to overcome the drawbacks of each technique and thus improve the accuracy of the diagnostic systems.

Similarly, Seccia et al. [15] reviewed studies that used computer-aided diagnosis (CAD) using clinical data alone or in conjunction with other forms of data to build prognostic models for MS. They pointed out some problems with the datasets used and recommended more collaboration between clinicians and computer scientists. Their findings imply that even though the number of publications in the field is huge, a clinically usable prognostic model for MS disease does not exist yet. 

Among the many benefits of DL and ML throughout the history of medicine, both can assist clinicians in the following: first in predicting those who are susceptible to the disease and hence alerting them regarding avoiding any triggers; second, in early and accurately diagnosing the disease, leading to utilizing therapeutic agents that are known to delay the prognosis of the disease and subsequently improving the quality of life of those patients; third, in predicting the transformation of the disease from one mild type to the other based on analyzing various blood, cerebrospinal fluid (CSF), and radiological markers; and fourth, in predicting the usefulness of certain medications in preventing the deterioration of the disease as well as treatment monitoring.

This paper provides a comprehensive review of the current literature studying different MS diagnosis techniques such as MRI, clinical data, and OCT using DL and ML. Most of the papers published since 2011 are organized and analyzed in a tabular form and examined from different viewpoints, including ML and DL models, dataset size, and performance. The keywords used to search for these papers are multiple sclerosis, diagnosis, machine learning, and deep learning. The main focus of this paper is automated MS diagnosis. However, a few progression papers have been included in this review as well. Moreover, the paper highlights some challenges and opportunities in the field of automated MS diagnosis.

The remaining part of this work is organized as follows: Section 2 presents numerous AI-based diagnosis approaches found in the literature. The most widely used algorithms and data types are discussed in Section 3. Finally, Section 4 concludes this paper.

## 2. Related Studies 

### 2.1. Machine Learning-Based Diagnosis Studies

Numerous studies were performed using ML techniques that are based on clinical symptoms or human activity data collected using sensors. Fiorini et al. [16] built a ML model to analyze clinical data for the detection of MS disease course. The aim was to distinguish between progressive and benign structures. The classifiers used were ordinary least squares (OLS), regularized least squares (RLS), K-nearest neighbors (KNN), logistic regression (LR), and linear SVM. Firstly, 91 features were collected from 457 patients. Then, missing values were imputed using the median. Afterward, a min-max scaling was used to normalize the dataset features fitting them into the [0:1] interval. The best accuracy obtained was 78.32% using the OLS algorithm with L1L2 feature selection. In addition, the highest F1-score of 70.2% was obtained using the RLS algorithm with L1L2 feature selection.

Similarly, Sarbaz et al. [17] aimed to develop a decision support system (DSS) that identifies MS patients relying on balance disorder using a noninvasive and simple method. That study enrolled 14 MS patients and 20 healthy controls. A marker was put on each participant’s forehead between the eyebrows. Then, participants were recorded while they stood in front of a black background for three minutes. The displacement of these markers was studied and analyzed using an image processing algorithm. An ANN was used with a ‘tan-sigmoid’ transfer function. Feature extraction depended on finding the features that were shown to be significantly different between the MS patients and the healthy controls. The ANN achieved an accuracy of 92.35%. Furthermore, the study authors developed another DSS that identifies people who are suspected of developing MS in the future and achieved an accuracy of 84.8%. These subjects who were classified as belonging to this intermediate state were recommended to refrain from being exposed to any MS triggers and to engage in activities that may prevent the onset of the disease. Specifically, they were recommended to consume appropriate amounts of vitamin D, avoid exposure to environmental and industrial toxins, and reduce stress.

Ettema et al. [18] examined the effectiveness of an electronic nose (eNose) in detecting MS based on exhaled breath analysis. This method was applied on 124 MS patients with a confirmed MS diagnosis and 129 healthy controls, who all breathed into the AeonoseTM for five minutes each. The volatile organic compounds in exhaled breath can be detected using the AeonoseTM diagnostic test device. AeonoseTM was tested to determine whether it could distinguish between healthy control subjects and patients with MS. Moreover, an ANN was trained using exhaled breath data. A second predictive model was created with a subgroup of MS patients without prescriptions for MS medications. According to the ANN model, MS patients could be distinguished from healthy controls with a sensitivity of 75% and specificity of 60%. The accuracy, sensitivity, and specificity of the model created with MS patients not on medication and healthy controls were 80%, 93%, and 74%, respectively. 

Lötsch et al. [19] proposed the creation of a complex serum lipid-biomarker classifier using supervised ML algorithms such as RF. The Bayesian statistics-based biomarker was trained using 403 patients to classify whether they were healthy or suffered from MS disease. Their clinical dataset was collected and preprocessed. In addition, RF was used to extract the most relevant features. The RF classifier trained with the complete feature set reached 100% sensitivity, specificity, and accuracy. However, a gap was observed between the ages of MS patients and the healthy subjects, and the data suffered from class imbalance.

Similarly, Martynova et al. [20] aimed to determine serum and CSF cytokine-based markers for MS diagnosis from a panel of 45 cytokines. CSF was gathered from 101 MS patients and 25 healthy controls. Cytokines were analyzed utilizing multiplex immunoassay. Furthermore, five ML models, namely, KNN, DT, XGB (XG Boost), Gaussian naïve Bayes (gNB), and RF were built by utilizing selected serum and CSF cytokines to diagnose MS and classify individuals into PPMS, SPMS, and RRMS. The features that were utilized as inputs to the ML models were selected based on ANOVA and on Pearson correlation coefficient scores; respectively, 22 and 20 cytokines were altered in CSF and serum. Based on a random selection of 5 biomarkers, the accuracy of MS diagnosis was ≥92% in all the experiments. Interestingly, an accuracy of 99% of MS diagnosis was achieved when CCL27, IFN-γ, and IL-4 were part of the 5 chosen cytokines. All five ML models exhibited relatively similar accuracy demonstrating that any of them could be utilized for MS prediction. Regarding classifying individuals into PPMS, SPMS, and RRMS, the XGB model reached an accuracy of 78% for serum, and the gNB model reached an accuracy of 69% for CSF.

Ali et al. [21] demonstrated a model that examined next-generation sequencing (NGS) data to derive MS biomarkers by inspecting transcriptomic microRNA data; it also integrates text mining approaches with ML methods for early MS detection. The dataset used was obtained from the National Centre for Biotechnology Information (NCBI) in the USA. It consists of next-generation sequencing (NGS) files of microRNA for 54 RRMS patients. An experiment was carried out on a transcriptomic dataset of MS patients prior to and after therapy with fingolimod, an immunomodulating medication. KmerFIDF was used for feature extraction, and linear discriminant analysis (LDA) was the dimensionality reduction method. Three classification models were applied, namely, RF, SVM, and LR. However, the RF algorithm outperformed other algorithms with sensitivity, specificity, F1-score, and average accuracy of 96.4, 96.47, 95.6, and 97%, respectively.

Acquaviva et al. [22] developed a ML pipeline using peripheral blood mononuclear cells (PBMCs). They built an unbiased framework based on nested cross-validation workflow comparing three ML algorithms: RF, functional trees (FT), and ADAboost-FT. The blood transcriptomes were acquired from 313 individuals: 60 healthy controls, 57 CIS subjects, 108 RRMS subjects, 26 SPMS subjects, 35 PPMS subjects, and 27 subjects with other neurological disorders. Several models were developed, each serving a different classification task. The first model differentiates between MS and non-MS cases. The second differentiates between CIS and HC, MS and the other neurological disorders. The last three models distinguish between PPMS and/or SPMS from RRMS. The ADAboost-FT outperformed the other algorithms in each scenario. In the MS vs. non-MS classification task, ADAboost-FT achieved 94.3% sensitivity and 87.5% precision, 77.8% specificity, and 88.7% overall accuracy.

Goyal et al. [23] developed a diagnosis model for MS using serum levels of eight cytokines, which are IL-1β, IL-2, IL-4, IL-8, IL-10, IL-13, IFN-γ, and TNF-α. They built several models including SVM, DT, RF, and ANN. For this study, 910 MS patients and 199 healthy controls were recruited, where 859 MS patients and 128 healthy subjects were from 2 American datasets, and 97 MS patients and 71 healthy controls were recruited from a Russian hospital. For the US data, Z score percentile method was applied, and 99.7% of the population was used for further analysis; 0.3% were excluded as outliers. Moreover, sixfold cross validation was applied three consecutive times to avoid bias. The RF achieved the best performance with regard to all metrics, with Gini score, AUC, accuracy, sensitivity, and specificity of 0.914, 0.957, 90.91%, 75.6%, 85.7%, respectively. Furthermore, another model for classifying the MS patients into remitting and non-remitting was built where the RF classifier achieved 70% accuracy. For the prognosis model, in addition to serum cytokines, age, gender, diseasesduration, EDSS and multiple sclerosis severity score were also included.

Sharifmousavi and Borhani [24] provide a simple and efficient method for detection of MS using vitamin D3, vitamin B12, and selenium levels. The serum levels of selenium and vitamins (B12, D3) in 99 MS patients and 81 healthy people were determined using atomic absorption spectroscopy and chemical autoanalyzer methods. In addition, three different supervised machine learning techniques, including SVM, DT, and KNN, were applied. The diagnostic model based on the SVM approach achieved thr best performance with an accuracy of 98.89%, sensitivity of 98.98%, positive predictive value of 98.98%, and true positive rate of 99.9%. 

Likewise, Pinto et al. [25] compared three ML models using SVM, KNN, DT, and LR. One for the prediction of conversion from RRMS to SPMS using clinical features obtained during the first five years of the disease, and two models for the prediction of disease severity after six and ten years. The study used a dataset from the Neurology Department of Centro Hospitalar e Universitário de Coimbra (CHUC) in Portugal. The dataset consisted of 187 patients for the MS conversion ML model, 145 patients for the disease severity prediction model in the 6th year after developing MS, and 67 patients for the disease severity prediction model in the 10th year. The dataset contained clinical data from MS patients suffering from RRMS and SPMS. For each prediction, five n-year models were built where a one year-model predicts using one-year clinical data from the progression of the disease. Feature extraction was applied to acquire the clinical data from the first N years since the patient’s first checkup in the clinic. After that, standardization, missing value imputation, and feature selection were applied to the data. Different patients were selected each time to be included in the training and testing sets, and this process was repeated 100 times. In these executions, the split of the training and testing sets was performed using ten different k-fold cross validations, each with a k value of ten. The final performance was identified by calculating the average values of all these executions’ results. Overall, SVM achieved the best results for the models. Since it is desirable to attain the least amount of data for the prediction, they considered the two-year model to have the best performance which achieved an AUC of 0.86 ± 0.07, sensitivity of 0.76 ± 0.14 and specificity of 0.77 ± 0.05. Regarding the sixth-year disease severity prediction, it was also desired to achieve good performance using data from the least number of progression years, the 2-year model was also chosen as the best predictor, reaching an AUC of 0.89 ± 0.03, sensitivity of 0.84 ± 0.11, and specificity of 0.81 ± 0.05.

Ashtiani et al. [26] proposed a ML method for classifying MS patients and healthy subjects via the most distinctive graph properties determined by statistical test and linear SVM classifier during the implementation of a cognitive task. The participants were 8 patients suffering from early stages of MS and 12 healthy subjects. Through the combination of all local measures, the node degree, subgraph centrality, K-Coreness, and PageRank centralities measured in the left fusiform, hippocampus, and parahippocampal gyri regions achieved an accuracy of 85%. Two optimal global measures, modularity and small-worldness index, and individual betweenness centrality enhanced the MS patient’s identification, achieving a sensitivity of 81.25%.

Kaur et al. [27] proposed a ML framework for recognizing MS using spatiotemporal and kinetic gait features after normalization. Gait data used in this study were gathered from 20 MS patients and 20 healthy older adults. Gait features were extracted from 3D ground reaction force data. The regression normalization increased the accuracy of identifying pathological gait utilizing ML compared with size normalization. As a result of generalizing from relaxed walking to walking while speaking, the gradient boosting (GB) algorithm reached the best subject classification with 94.3% accuracy, 1.0 AUC, and 1.0 precision. However, for subject generalization, a multi-layer perceptron (MLP) reached 80% accuracy and 0.86 AUC with regression-normalized data.

Lim et al. [28] proposed a method for studying the association between inflammation, the kynurenine pathway (KP), and MS pathogenesis as they identified that serum KP metabolic signatures in patients can be used to distinguish clinical MS subtypes with high specificity and sensitivity. Four classifiers, namely, regression tree, SVM, discriminant analysis, and C5.0 DT, were used in the study. The best-performing model was the C5.0 DT classifier, which was trained with data collected from 136 participants consisting of 50 RRMS, 17 PPMS, 20 SPMS, and 49 healthy controls. The model successfully classified the clinical subtypes of MS with a sensitivity of 91%. In addition, they performed another independent study using data collected from 10 patients with RRMS, 20 patients with SPMS, and 6 healthy controls, and the model’s sensitivity was maintained at 85%. 

Mezzaroba et al. [29] aimed to evaluate indicators of MS disease in order to enable MS diagnosis. The study included 174 MS patients and 182 healthy controls. The findings showed that MS is associated with a decrease in levels of zinc, total radical-trapping antioxidant parameter, adiponectin, and sulfhydryl and increased levels of advanced oxidation protein products. They used an SVM classifier with 10-fold cross validation and obtained an accuracy of 90.6%.

Hu et al. [30] incorporated ML algorithms used on raw walkway data to distinguish between MS patients and healthy controls. They focused on constructing a series of novel features to enhance standard parameters which in turn improves the model’s performance. Hence, they used an instrumented walkway to generate rich data that are usually unnoticed by clinicians. The data were collected from 72 MS patients and 16 healthy controls. They selected 11 features of which 5 were novel supplementary features and trained their SVM classifier. The model achieved an accuracy of 81%, sensitivity of 81%, precision of 95%, and F1-score of 87%. 

Another interesting batch of studies was conducted to diagnose MS using MRI features. Elliott et al. [31] suggested a method that segments sequential scans jointly for providing an accurate temporally consistent segmentation of tissue while preserving sensitivity to newly emerging lesions. This method was applied on 364 MRI scans taken from 95 patients from a multicenter clinical trial. The approach involves two stages of the classification process: a Bayesian classifier, which gives a potential brain tissue grouping for every voxel of reference and scans, and a RF for the recognition of newly emerged lesions. In addition, 63 features were found. Voxel-wise classification was utilized for feature selection and revealed that the most valuable feature was the mean probability of a new lesion:. For new lesions that were of size greater than 0.15 cc, the classifier achieved a 99% sensitivity and 2% false detection rate.

Zhang et al. [32] proposed a novel MS identification approach from brain MRI. The dataset used was collected from 38 MS patients obtained from the eHealth lab at the University of Cyprus and 34 healthy controls obtained from China’s local hospitals. The data imbalance was handled through applying synthetic minority oversampling technique (SMOTE). After that, distinguishing edges were extracted utilizing canny edge detector. Feature extraction from edges was achieved with the Minkowski–Bouligand dimension (MBD). The classifier used was a single hidden-layer neural network. To train the classifier, three-segment representation biogeography-based optimization was employed. The proposed approach reached sensitivity, specificity, and accuracy of 97.78 ± 1.29%, 97.82 ± 1.60%, and 97.80 ± 1.40%, respectively.

Similarly, Wang et al. [33] aimed to find a method of detecting the early phases of MS. They used 676 MRI slices holding plaques of 38 patients and 880 MRI scans of 34 healthy people. They proposed a new classifier method based on three techniques, which were biorthogonal wavelet transform (BWT), radial basic function kernel principal component analysis (RKPCA), and LR. They used discrete wavelet transform (DWT) to extract the features. Then, they utilized a principal component analysis (PCA), which is an efficient dimensionality reduction tool, to diminish the size of wavelet coefficients of brain MRI. Kernel PCA (KPCA) was used to overcome the weakness of PCA as is it cannot extract nonlinear structure data. Furthermore, binary LR with ten-fold cross-validation was utilized to train the model. The study achieved sensitivity of 97.12 ± 0.14%, specificity of 98.25 ± 0.16%, and accuracy of 97.76 ± 0.10%.

Correspondingly, Zhang et al. [34] used MRI to recognize MS subjects from healthy controls. This study utilized scans for 38 MS patient downloaded from the eHealth laboratory at the University of Cyprus and 38 healthy subject controls imaging data obtained from volunteers in their local hospital. Two-level stationary wavelet entropy (SWE) was used to extract features from MRIs. Then, they used three classifiers which are DT, KNN, and SVM. The SWE + KNN achieved the highest accuracy of 97.94%, specificity of 99.32%, and sensitivity of 96.15%. 

Likewise, Zhang et al. [35] predicted whether CIS will converge into MS by analyzing the MRI image features of the lesions. The study was performed on 84 patients diagnosed with CIS. McDonald criteria were used to determine conversion to MS. Three-dimensional FLAIR and three-dimensional T1 images were used to segment brain lesions. A computer-assisted manual segmentation system was used to generate lesion masks. Moreover, the Lesion Segmentation Toolbox for SPM was also used to generate automated segmentations to test the effectiveness of different segmentation methods. The segmented masks were automatically used to calculate shape and brightness features, which were also used as input data for training an oblique RF classifier. The classifier achieved accuracy of 84.5%.

Saccà et al. [36] performed a comparative analysis of several ML techniques to identify which method would prove most effective for early diagnosis of MS. The study recruited 18 MS patients and 19 healthy controls from the Neurological Unit of the University Magna Graecia of Catanzaro Italy. An independent component analysis (ICA) network dataset was analyzed using RF, SVM, NB, KNN, and ANN algorithms. Then, each classifier’s features were selected, and the results were compared. Both SVM and RF demonstrated the same accuracy of 85.7% and the same specificity of 66.7% using 5-fold cross-validation.

Moghadasi et al. [37] aimed to classify MS patients based on MRI scans. They demonstrated that 3D images can be transformed to 2D images using SVM tools as 2D images are more efficient at handling ML processing. The 72 brain MRI scans were examined by applying an SVM classifier. Four models were built using one-against-all (1AA) and six models were built using one-against-one (1A1). The 1AA classifier achieved an average accuracy of 77.83% whereas the 1A1 achieved an average accuracy of 76.52%.

Similarly, Rezaee et al. [38] proposed a hybrid automatic processing technique for MS detection based on features extracted from MRI scans. The data were privately collected over a period of 18 months from 64 patients with different levels of MS at the Vasei Hospital Iran and 61 healthy subjects. Fractal and pseudo-Zernike moments (PZM) methods were used for feature extraction to create a feature vector of slices, and feature selection was performed using the differential evolution (DE) algorithm. The algorithm used was ELM with its wavelet kernel parameters optimized using the shuffled frog-leaping algorithm (SFLA), and the average accuracy obtained was 97% using 5-fold cross validation.

Ekşi et al. [39] used a CAD method to distinguish MS from low-grade brain tumors using magnetic resonance spectroscopy (MRS) data on 51 MS patients and 39 low-grade brain tumor patients. Feature extraction was carried out using the peak integration and full-spectrum techniques to identify the most significant features in MRS data. ANN, SVM, and LDA were used for classification. They found that the ANN-based system was able to differentiate brain tumors and MS signals from MRS signals with accuracy of 100%, specificity of 100%, and sensitivity of 100%. However, the study used a small sample size of only 90 records.

Peng et al. [40] aimed to use radiomics model to predict the progression of unenhanced MS lesions on fluid-attenuated inversion recovery (FLAIR) images and to investigate its optimal model. For data collection, 45 MRI scans were obtained from 36 MS patients. Radiomics features of lesions were extracted from FLAIR images. For feature selection, recursive feature elimination (RFE), ReliefF algorithm, and least absolute shrinkage and selection operator (LASSO) were used. In order to create predictive models, three ML classifiers were used: logistic regression (LR), RF, and SVM. Nine models were created and evaluated based on the combinations of three ML classifiers and three feature selection algorithms. The best prediction results were acquired with the SVM classifier using the ReliefF algorithm, with average accuracy, sensitivity, specificity, and AUC of 82.7%, 80.9%, 84.1%, and 0.857, respectively.

Similarly, Eshaghi et al. [41] aimed to categorize MS disease types using clinical features by applying unsupervised ML using MRI scans. In this study, they used Subtype and Staging Inference (SuStaIn), an unsupervised ML algorithm they developed [42]. The model was trained to classify MS patients into the four phenotypes using a dataset consisting of 6322 MS patients, and a different group of 3068 patients was used for validation. Furthermore, to normalize the dataset, an internal reference was used, the CSF that fills the ventricles of the brain. Despite aiming to use MRI data instead of relying solely on clinical data, it was found that combining both increased the prognostic accuracy of the model.

Elsebely et al. [43] introduced a hybrid ML model to solve two problems: MS lesion detection and handling imbalanced data without loss using a cost function. The dataset was obtained from an MS lesion segmentation challenge 2008 workshop. Two-dimensional discrete wavelet transforms (2DD WT) and textural features were used for feature extraction from MRI scans. An ensemble ML model was developed for MS detection using textural features. The best result was obtained using ensemble SVM (ESVM) and ensemble decision tree (EDT). The model achieved accuracy of 98.2% for ESVM and 98.5% for EDT.

Similarly, Merzoug et al. [44] developed an approach for MS diagnosis using a segmentation technique for the detection of MS lesions in MRI scans. This method was built using artificial immune systems (AIS) and SVM with RBF kernel. Based on their model, AIS was used to separate the brain tissues into three segments. After feature extraction, an SVM model that was based on sequential minimal optimization algorithm (SMO) was used to classify MS lesions. The proposed approach achieved accuracy, sensitivity, and specificity of 99.8%, 100%, and 83.8%, respectively.

Likewise, Aoki et al. [45] aimed to build a ML model that classified subjects into healthy, PRMS patients, and PPMS patients based on quantitative measures for brain atrophy features caused by MS. The dataset contained brain volumes obtained from 55 segments of the brain region calculated from MRI scans. The MRI scans were acquired from 72 MS patients and 21 healthy controls from the Department of Neurology at Tohoku Medical and Pharmaceutical University Hospital in Japan. The authors performed preprocessing techniques including automated segmentation and normalization. Moreover, they performed a logarithmic conversion for segments that were in a lognormal distribution. They used two classifiers, Bayesian regularized neural networks (BRNN) and SVM algorithms, and conducted experiments using different numbers of brain segments, namely 55 and 15. The top 15 segments produced better results for the BRNN classifier. The BRNN method achieved 77.8% sensitivity, 95.2% specificity, and an AUC of 0.904.

Bonanno et al. [46] developed a CAD system using a hybrid watershed-clustering algorithm for automating image segmentation to distinguish MS lesions from non-lesions. For the dataset, the MR images of 20 MS patients were analyzed. A watershed algorithm was applied to identify the structures within the MS lesion, utilizing adaptive filters to improve the structures within the lesion. Furthermore, a set of meaningful features were estimated on each region of interest (ROI) extracted from each MR image based on the detected MS lesions. Cluster analysis was used to solve the problem of unwanted over-segmentation resulting from the watershed algorithm. The proposed method achieved diagnostic accuracy of 87%, sensitivity of 77%, and specificity of 87%.

Iswisi et al. [47] developed a ML model for MS diagnosis based on the Harris Hawks optimization (HHO) algorithm using MRI scans of 10 patients. The fuzzy C-means (FCM) algorithm was combined with the HHO algorithm for the extraction of lesions and reduction of the segmentation error. Moreover, the HHO algorithm was used to choose the cluster centers for the purpose of detecting MS lesions. For the population of the HHO algorithm, the membership matrices were selected that are used to obtain the optimal cluster centers. In addition, the HHO algorithm was utilized to select the optimal membership matrix based on the chosen cluster centers, for accurate segmentation and detection of MS lesions. The final results revealed that the use of the proposed model on images indicates that using three cluster centers yields to excellent results in the segmentation of MRI scans. The method achieved an accuracy, sensitivity, and specificity of 94.23%, 89.56%, and 93.34%, respectively.

A comparative analysis of several ML classifiers on 18 gray-level textural feature matrix (GLTFM) of MRI scans was performed by Jain et al. [48]. They used classification models such as KNN, SVM, and ensemble learning and then compared them with unsupervised techniques including k-mean clustering and Gaussian mixture model. The MRI scans were collected from two datasets: 110 healthy MRI scans ware privately collected, and 82 MS scans were obtained from the e-health lab dataset. They concluded that supervised ML techniques outperformed unsupervised ML techniques in distinguishing between healthy subjects and MS patients. The KNN classifier and SVM with polynomial kernel achieved the highest accuracy of 96.55%.

Han and Hou [49] proposed a classification method based on wavelet entropy and feedforward neural network that was trained using an adaptive genetic algorithm (AGA). The dataset used contained 676 MRI slices from 38 MS patients obtained from eHealth Lab, and 681 MRI slices from 26 healthy controls. Since these two datasets were obtained from different sources, histogram stretching was used to normalize them and achieve inter-scan normalization. Their method used wavelet entropy, ANN, and AGA, where the feature extraction was achieved using the wavelet entropy, classification was performed by the ANN, and the AGA was used as a training algorithm to benefit from its capability of global optimization. The approach was implemented over 10 runs of 10-fold cross validation. The best performance was obtained using wavelet decomposition level of 3, which achieved sensitivity, specificity, precision, and accuracy of 91.91% ± 1.24%, 91.98% ± 1.36%, 91.97% ± 1.32%, and 91.95% ± 1.19%, respectively.

Wu and Lopez [50] proposed a novel MS slice identification system, based on Haar wavelet transform, PCA, and LR. The dataset was obtained from local hospitals in China and contained 141 MRI slices from 34 MS patients and 148 slices from 33 healthy controls. The model achieved the highest accuracy of 89.72 ± 1.18% using 3-level Haar decomposition.

Azarmi et al. [51] aimed to build a model using linear SVM, polynomial SVM, and regression to differentiate between MS patients and healthy controls using brain network features. They used graph theory and task-related fMRI data obtained from early stages of the disease. fMRI data was obtained from 20 individuals, 8 RRMS patients from Firoozgar Hospital in Tehran, Iran, and 12 healthy controls. The most important features were chosen using a combination of Wilcoxon rank-sum test and Fisher score. The linear SVM achieved the highest accuracy of 95% when using 8 or 9 features. It achieved 87.5% sensitivity. All models achieved 100% specificity.

Macin et al. [52] introduced a handcrafted feature engineering approach to construct a computationally lightweight ML model for MS diagnosis. The dataset used in the study consisted of axial and sagittal brain MRI scans that were collected from 72 MS patients and 59 healthy controls. They performed three experiments using three subsets of the data. Moreover, to generate the features they used a fixed-size patch-based (exemplar) feature extraction model based on local phase quantization (LPQ) producing the Exemplar Multiple Parameters LPQ (ExMPLPQ) features. These features were combined to produce a large final feature vector. Iterative neighborhood component analysis (INCA) was used for feature selection. They finally trained their KNN classifier to distinguish between MS patients and healthy controls. The ExMPLPQ-based KNN model with 10-fold cross validation achieved an accuracy of 98.37% using axial images.

Neeb and Schenk [53] analyzed the performance of different multivariate supervised ML models in diagnosing MS using features derived from quantitative MRI scans. The data was collected from 52 MS patients and 45 healthy controls. They focused on enabling diagnosis even through images degraded due to motion. Their model achieved an accuracy of 83.7% when using data that was not affected by motion. However, when MRI scans of degraded quality due to motion were included, the accuracy achieved was reduced to 74.5%.

Zurita et al. [54] aimed to classify RRMS patients and healthy controls through MRI scans using SVM. The model had four input features, structural and functional connectivity, fractional anisotropy maps, and a combination of structural and functional connectivity. Furthermore, the Fisher criteria were used as a dimensionality reduction technique. The dataset consists of 104 RRMS patients and 46 healthy controls. The RRMS patients were further divided into two groups based on the degree of disability. The binary classifier reached accuracy of 88.9% ± 2.4%. On the other hand, the multiclass classifier acheived accuracy of below 63% ± 5%.

Deshpande et al. [55] proposed a method for classifying MS lesions using sparse representations and dictionary learning. It was shown that learning more detailed dictionaries for anatomic structures in the brain resulted in improving performance, due to specified intensity patterns related to the structures that are found in multi-channel MRI. Furthermore, the approach shows that adapting the dictionary sizes can also improve classification results. The method achieved a sensitivity of 99.5% and PPV of 2.1.

Despite lesions being the most releasing indicator of MS, Yoo et al. [56] introduced a method of diagnosing the disease based on measuring myelin content. They used myelin imaging, which is a quantitative form of MRI scans used to identify and assess myelin content that can possibly enable the diagnosis of MS at an early stage. They proposed a ML model that is trained on extracted 3D image patches from myelin maps and their associated T1-weighted MRIs. The study included 55 RRMS patients and 44 healthy control patients. They performed a voxel-wise t-test between the two sets of patients to select a common set of images. For feature selection, they used LASSO to select normal-appearing features to construct an RF classifier using 11-fold cross validation. The model obtained average classification accuracy of 87.9%, specificity of 88.6%, sensitivity of 87.3%, and AUC of 0.88.

Other studies combined more than one data type in their data collection process for the classification of MS patients. Bejarano et al. [57] aimed to predict the short-term prognosis of MS. A prospective cohort study was performed on 51 MS patients and 20 healthy controls in San Raffaele Hospital in Italy. In the study, motor evoked potentials (MEP), MRIs, and clinical data were gathered from the patients. The classifiers used were random decision trees, Bayesian, simple LR, and NN. The models’ goal was to predict disability progression, Expanded Disability Status Scale (EDSS) score, and new relapses. Moreover, an adjusted protocol for enhanced voxel-based morphometry (VBM) with optimizations specifically for MS was used to normalize and segment images while avoiding bias in addition to using the Wrapper approach for attribute selection. To validate the model, 10-fold cross validation was applied along with conducting a 2nd cohort study including 96 MS patients from a different center. The best performance achieved was accuracy of 80% for detecting EDSS change two years ahead.

Kocevar et al. [58] used demographic, clinical data, and MRI to build an SVM with a radial basic function (RBF) kernel to classify patients into the four clinical groups of MS. The experiments were performed on 64 MS patients, and the acquired data were preprocessed included correction using Eddy current and non-brain voxels stripping. In addition, for parameter tuning grid search was used on the two SVM parameters to reduce the likelihood of biases, K-fold cross-validation was used to enhance classification results. The highest obtained F1-score for classifying MS was 91.8% for HC-CIS, CIS-RR classification.

Moreover, Zhao et al. [59] intended to show the significance of ML in detecting MS progression. The study classified patients as worsening or non-worsening cases using SVM and LR classifiers. A comprehensive longitudinal investigation of MS was performed at the Brigham and Women’s Hospital Boston (CLIMB) to obtain demographic, clinical, and MRI data from 1693 patients. A semi-automated template-driven segmentation tool was used to process all the scans, and whole-brain volume was normalized. The features were analyzed with regard to their contribution, whether positive or negative, to developing an understanding of the most relevant features to each class. The highest results achieved were accuracy of 70%, sensitivity of 71%, and specificity of 68%.

In the same manner, Ion-Margineanu et al. [60] utilized three classifiers, LDA, RF, and SVM with radial base function (SVM-RBF), to classify patients into one of the four MS subtypes. A study was performed on 87 MS patients, and the authors collected lesion loads combined with clinical data, MR metabolic features, and a total of 592 scans. Precise quality control was used for the extraction of metabolic features. The dataset suffered from imbalance that was handled by resetting the parameters for each classifier. The LDA was tuned using shrinkage and selection methods. The SVM was tuned using a logarithmic grid search; for the RF classifier, the number of DT was tuned. The highest F1-score of 87% for RR vs. SP was achieved with SVM-RBF and LDA trained using a combination of all the data collected.

Another approach was proposed in several studies to use other types of data such as various retina features and different types of evoked potentials (EP) to train the models. Palomar et al. [61] proposed a model for the early prediction of MS using swept-source optical coherence tomography (SS-OCT) data. Average retinal nerve fiber layer (RNFL) and complex ganglion cell layer–inner plexiform layer (GCL+) thickness measurements were obtained from 80 patients with RRMS and 180 age-matched healthy controls. The RNFL data proved to be the best for the prediction of the disease. A ten-fold cross-validation resampling method was applied. The best result was achieved using a combination of DT and AdaBoost (AB) algorithms with accuracy of 97.24%.

In the same way, Cavaliere et al. [62] developed an SVM with a Gaussian quadratic kernel for the diagnosis of MS by utilizing structural neurodegeneration in the retina. In their study, the dataset consisted of 48 MS patients and 48 healthy controls. SS-OCT was applied on OCT data to fetch the values for macular thickness and peripapillary area. Feature selection was applied, and three variables with the highest discriminant capacity were identified and used. The variables that were sufficient to classify MS patients were GCL++_Total (global GCL++ thickness evaluated at the peripapillary area), ETDRS_ON_Retina (macular retina thickness in the nasal quadrant of the outer ring), and ETDRS_IN_Retina (macular retina thickness in the nasal quadrant of the inner ring). The model achieved an accuracy of 91%.

Similarly, Garcia-Martin et al. [63] introduced a method for early diagnosis of MS based on the analysis of retinal layer thickness acquired using swept-source optical coherence tomography (SS-OCT). In this study, OCT recordings were collected from 48 newly diagnosed MS patients and 48 healthy controls. In a 45 × 60 grid, the thicknesses of the retinal nerve fiber layer (RNFL), the ganglion cell layer (GCL+), the GCL++, and the retinal thickness were determined. The Cohen’s d effect size was utilized to specify the regions and layers with the greatest capacity to differentiate between control subjects and patients. The points that exceeded the chosen threshold were used as inputs for the automatic classifier. They used SVM and feed-forward neural network (FFNN) classifiers. GCL++ shows the highest discriminant capacity at the onset of MS with an AUC of 0.83, which exhibits a horseshoe-like macular topographic distribution. The FFNN had the best performance and achieved sensitivity of 98%, specificity of 98%, and accuracy of 98%.

Likewise, Montolío et al. [64] used OCT to measure patients’ RNFL thinness for MS diagnosis and prognosis using ML techniques. The study included 72 MS patients and 30 healthy controls, and the classifiers used in this study included SVM, Multiple Linear Regression (MLR), KNN, DT, NB, ensemble classifier (EC), and long short-term memory (LSTM-RNN). To perform feature selection, LASSO and sequential forward selection (SFS) were used to remove the irrelevant features. For MS diagnosis, the best results were obtained using KNN, which achieved accuracy of 95.8%, sensitivity of 94.4%, specificity of 97.2%, precision of 97.1%, and AUC of 0.958. As for MS prognosis, the best results were obtained using the DT classifier, which achieved accuracy of 91.3%, sensitivity of 90.0%, specificity of 92.5%, precision of 92.3%, and AUC of 0.913.

Similarly, Kenney et al. [65] suggested using ML classification to set thresholds for OCT inter-eye differences (IEDs) to aid in MS diagnosis. They measured the peripapillary RNFL and ganglion cell + inner plexiform (GCIPL) thicknesses using spectral-domain optical coherence tomography (SD-OCT). The dataset was composed of 1568 MS patients and 552 healthy controls. The SVM classifier was used and achieved accuracy of 81%, sensitivity of 81%, specificity of 80%, and an AUC of 0.89. 

L’opez-Dorado et al. [66] developed a CAD system to detect MS disease based on analyzing the outer retina as assessed by multifocal electroretinograms (mfERGs). To analyze the outer retina, 21 scans were taken from patients. Considering the small size of the dataset, the binary SVM classifier was used in this study because it is less affected by sample size. Initially, 40 features were collected. However, using a wrapper-feature selection and a filtering method, the four most relevant features were extracted. For preprocessing, statistical analyses were performed followed by an analysis of normality to prepare the data. Finally, with the optimal CAD configuration, accuracy of 95%, specificity of 100%, and sensitivity of 93% were obtained.

Mohseni and Moghaddasi [67] introduced a hybrid approach for MS diagnosis with an aim to decrease the classification error rate. In the study, they focused on analyzing Electroencephalogram (EEG) descriptors in both the time and frequency domains. The study included 19 MS patients and 21 healthy controls. After feature extraction, an enhanced version of ant colony optimization was used for feature selection. Then, the SVM algorithm was used for MS diagnosis using wavelet analysis techniques and signal windowing and dividing all five subbands of EEG signals. The highest accuracy level achieved was about 99.03%, a sensitivity of 98.90%, and a specificity of 99.18%.

Likewise, Ahmadi et al. [68] developed a CAD system that uses EEG signals to diagnose MS using online sequential extreme learning machine (OSELM). The study was conducted with five MS patients and seven healthy participants. The EEG signals were recorded from both groups while applying covert visual attention to both the color and direction. To select informative features, T-test and Bhattacharyya distance criteria were applied. The classifier achieved accuracy, sensitivity, and specificity of 91%, 83%, and 96% for the color task, and 90%, 82%, and 96% for the direction task, respectively.

Karaca et al. [69] aimed to diagnose MS in male patients using photic stimulation electroencephalogram (PS EEG) signals. The data were collected from 20 male subjects. Initially, they used photic stimulation and applied continuous wavelet transformation (CWT) to EEG signals with five frequencies ranging from 5 Hz to 25 Hz. Afterwards, several ML models were evaluated, and the ensemble subspace KNN model obtained the highest accuracy, sensitivity, and specificity of 80%, 72.7%, and 88.9%, respectively. Table 1 contains the summary of the MS diagnosis studies using machine learning. The table contains the testing results achieved by the proposed models.

Santiago et al. [70] aimed to classify individuals into the different stages of MS using multifocal visual evoked potentials (mfVEPs). The dataset included 96 individuals classified into patients with radiologically isolated syndrome (RIS), patients with CIS, definite MS patients, and healthy controls. The study proposed a flat multiclass classifier (FMC) and a hierarchical classifier (HC), where both were built applying the KNN algorithm. In this system, the eyes are classified first according to their mfVEP recordings and consequently; the diagnosis is performed on the subjects. The HC achieved the highest eye and subject classification accuracy of 74% and 95%, respectively. 

Similarly, Yperman et al. [71] proposed another approach to predicting the disability progression of MS patients after two years using an LR classifier and an RF classifier with a 100 DTs and balanced class weights. The study utilized EPs obtained from the Rehabilitation & MS Center in Overpelt, Belgium. The authors investigated the MEPs of 642 patients. They extracted a huge number of time-series features from these MEPs. The top features were selected based on mutual information with the target and the Boruta method. The RF achieved the best performance with an AUC of 0.75 ± 0.07.

Solana et al. [72] aimed to develop a model that could classify individuals into MS patients and healthy controls using structural brain connectivity features. They identified 42 features from the properties that best defined the two groups, which are node strength and local efficiency, to build an SVM with k-fold cross validation. For this study, 45 healthy individuals and 188 MS patients were recruited from the MS Unit of the Hospital Clinic of Barcelona, and random undersampling was applied for the majority class. Their method achieved accuracy of 77.15% for local efficiency and 74.84% for node strength. Their findings suggest that central network properties of vulnerable nodes can discriminate MS patients from healthy controls.

Kawahara [73] aimed to predict MS disability using spinal cord features. They used MRI and the segmentation of the spinal cord that are related to the clinical status to extract new features. By utilizing the extracted features, they applied various regression models such as simple LR models, multiple LR models, and non-linear non-parametric RFs. To decide which features were beneficial biomarkers, they examined the features’ data that were related to the clinical status. The results showed that examining the length between the cord’s center-of-mass and the cord’s boundary feature gave the best results and was an advancement at clinical prediction over the volume of the spinal cord. The RF obtained the lowest mean absolute error (MAE) of 0.293 and root mean squared error (RMSE) of 0.353.

### 2.2. Deep Learning-Based Models

Some studies have used DL techniques for the diagnosis of MS using clinical data or human activity data collected via several sensors. Casalino et al. [74] developed a multi-class classification model that discriminates between ADHD and pediatric MS using miRNA expressions. They experimented with RF, extremely randomized trees, and multi-layer perceptron (MLP). The dataset included expressions from 1287 miRNAs obtained from 47 children participants, where 20 were healthy controls, 19 suffered from pediatric MS, and 8 had ADHD. Data preprocessing included normalization, feature selection, and oversampling. Three feature ranking techniques were overlapped to produce a robust selection of 40 significant features. The MLP achieved accuracy of 81% using 5-fold cross-validation.

In a similar manner, Schwab et al. [75] aimed to introduce a DL method that diagnoses MS from the smartphone-derived digital biomarkers. The data was collected from 774 participants. The study utilized data obtained from the Floodlight Open study, a huge smartphone-based observational study of MS. Participants of this study were requested to conduct every day on their smartphones several tests without any clinical supervision. The authors utilized an attentive aggregation model (AAM) to aggregate data from various test types over a lengthy duration to generate a scalar diagnostic score. They found that overall AAM + age + sex achieved the best results with a sensitivity of 83%, F1-score of 80%, and an AUC of 0.88. However, the mean aggregation model obtained a higher specificity of 85%. So, digital biomarkers obtained from smartphone data could be utilized as extra diagnostic measures for MS in the future.

While several studies utilized MRI scans for the diagnosis, La Rosa et al. [76] compared deep and shallow learning architectures for the automated segmenting of white matter lesions in MRI for people with MS. The study was performed on 34 patients. Two recent MS segmentation methods were chosen. In the first step, the partial volume (PV) modeling combined with supervised KNN technique, developed especially for subjects who have a low disease burden and small lesions. Secondly, using a newly existing DL algorithm using two 3D patch-wise CNNs. Results were compared between LeMan-PV, CNNs, and PV-CNNs strategies. The following evaluation metrics were calculated according to three MS lesion segmentation challenges: lesion-wise false positive (LFPR) and lesion-wise true positive rates (LTPR), overlap dice coefficient (Dice), voxel-wise true positives (TP), and volume difference (VD). The best segmentation results were obtained by LeMan-PV with the highest dice coefficient of 63% and the smallest volume difference of 19%. CNNs had the lowest LFPR of 30%. Moreover, a grouping of the two methods PV-CNNs improved their LFPR of 26%, LTPR of 69%, but perform poorly in the VD.

Similarly, Eitel et al. [77] developed a transparent DL framework based on CNN and layer-wise relevance propagation (LRP) for MS diagnosis. The MRI scans were provided by FP from Charite–Universit’ atsmedizin Berlin for VIMS study, with a sample size of 147 patients. PCA was utilized for dimensionality reduction, LRP for feature extraction, and grid search for hyperparameters tuning. The framework analyzed neuroimaging records using CNN, which aids in illustrating separate classification decisions. Remarkably, a pretrained CNN could diagnose patients with MS, with an accuracy close to a classic ML algorithm. In addition, LRP visualization showed that the CNN model not only considered individual lesions but could also detect extra information like lesion location, non-lesional white matter, and grey matter areas, which all represent MRI markers in MS. The CNN model achieved an accuracy of 87.04%, specificity of 81%, sensitivity of 93.08%, and an AUC of 0.9608.

Sepahvand et al. [78] used a convolutional neural network (CNN) to detect MS lesions using subtraction images on 1677 MRIs collected from 886 MS patients. For cross-validation, the training set was further divided into fivefold. Moreover, preprocessing included brain extraction, correction of bias field inhomogeneity, registration of all images to MNI-space, normalization of Nyul image intensity, and rescaling all the scans to the [0:1] range. The CNN classifier reached overall accuracy of 95%, specificity of 97%, and sensitivity of 69%.

Similarly, Roca et al. [79] proposed a model for predicting the EDSS using sex, age, and FLAIR MRI data for patients with MS. For the study, 971 MS subjects were used to train the model obtained from the Observatoire Franc¸ ais de la Sclérose En Plaques (OFSEP) cohort dataset, consisting of FLAIR MRI with EDSS score. The EDDS score was removed from the test set consisting of 475 subjects. Furthermore, Adam optimizer was used for parameter tuning, and dimensionality reduction was implemented using handcrafted features with 65 features. The study used CNN, RF regressors, and a manifold learning algorithm that uses the location of lesion loads on white matter tracts. As for the results, MSE = 2.2 for the validation dataset and an MSE = 3 (mean EDSS error = 1.7) for the test dataset were accomplished.

In the same manner, Soltani et al. [80] proposed methods for improving the CNN classifier for MS disease detection using MRI. The proposed model consisted of seven layers and was employed for feature extraction and classification. The model included four layers of convolution and three layers of rectified linear unit (ReLU). An extra two layers of max pooling were used to cut down the size of the image to reduce the number of parameters and calculations. Moreover, the model used a convolution layer with a filter instead of fully connected, condensed network parameters. The study utilized the MRI from a database of 72 patients. These images were preprocessed by converting the three-dimensional images into grey images and unifying their size. It was noted that CNN did not require lesion segmentation and nor was it sensitive to blurring and different contrast. Hence, it was concluded that CNN was a promising technique for the diagnosis of MS disease as it achieved 99.66% accuracy, 99.33% specificity, and 99.98% sensitivity.

Siar et al. [81] aimed to utilize CNN for simultaneous diagnosis of a brain tumors and MS. The MRI dataset was collected from 200 subjects, including brain tumors, MS, and healthy subjects. Comprehensively, there were 461 images for the brain tumor patients, 791 images for the healthy controls, and 320 images for the MS patients. The result of the proposed method on 384 test data achieved an accuracy of 96.88%.

Wang et al. [82] introduced a six-layer stochastic pooling CNN to detect MS with multiple-way data augmentation. The MRIs dataset utilized was collected from 38 MS patients acquired from the Laboratory of eHealth of the University of Cyprus and 26 healthy controls acquired from a private source. In order to assess the impact of stochastic pooling and multiple-way data augmentation to the original CNN model, ablation experiments were performed. The sensitivity, specificity, and accuracy of the introduced approach were 95.98 ± 0.46%, 95.67 ± 0.92%, and 95.82 ± 0.58%, respectively.

Wang et al. [83] introduced a 14-layer CNN with batch normalization, dropout, and stochastic pooling. The MRI dataset used was collected from 38 MS patients obtained from the Laboratory of eHealth of the University of Cyprus and 26 healthy controls obtained from a private source. By activating the pooling regions, a multinomial distribution was constructed and sampled to obtain the outcome of stochastic pooling. Batch normalization and dropout were used to solve the issues encountered in the traditional CNN, including internal co shift invariant and overfitting. Moreover, the training set was enhanced by applying data augmentation. This method achieved sensitivity, specificity, and accuracy of 98.77 ± 0.35%, 98.76 ± 0.58%, and 98.77 ± 0.39%, respectively.

Zhang et al. [84] aimed to develop a ten-layer CNN model incorporating the parametric rectified linear unit (PReLU) and dropout techniques for MS identification. The dataset utilized was collected from two different sources. The MS MRIs were collected from 38 MS patients obtained from the Laboratory of eHealth of the University of Cyprus. In addition, the healthy MRIs were collected from 26 healthy controls obtained from a private source. Moreover, the training set was expanded by utilizing data augmentation. The ten-layer CNN model includes seven convolution layers and three fully connected layers. The 3 dropout layers’ retention probabilities were 0.4, 0.5, and 0.5, respectively. Finally, the proposed approach reached sensitivity, specificity, and accuracy of 98.22%, 98.24%, and 98.23%, respectively.

Yılmaz Acar et al. [85] developed a CNN model for MS diagnosis through the detection of lesions in brain FLAIR MRI. The dataset utilized consist of brain MRI, brain mask, and ground truth data of 30 MS patients obtained from the Laboratory of Imaging Technologies (LabIT). MS lesions features in MRIs are extracted with a small set of trainable parameters. The results were produced from data splitting at slice level as well as at patient level. Using slice-level splitting, the proposed model reached an accuracy, sensitivity, specificity, and precision of 98.0 ± 0.02%, 97.9 ± 0.03%, 98.3 ± 0.03%, and 98.2 ± 0.03%, respectively. Using patient-level splitting, the proposed model reached accuracy, sensitivity, specificity, and precision of 90.3 ± 0.05%, 90.5± 0.05%, 90.1± 0.09%, and 91.1± 0.09%, respectively.

Fooladi et al. [86] compared three ANN-based models, including MLP, RBF, and ensemble neural networks based on Akaike information criterion (ENN-AIC). The MRI dataset of 30 healthy controls and 30 RRMS patients was collected from the neurological research center of Tehran University of Medical Science. Using parametric maps, the input features were extracted as the average values of quantitative magnetization transfer imaging (QMTI) and T1.The outcomes show that the ENN-AIC model outperformed the other ANN models with an accuracy of 90%, sensitivity of 92%, and precision of 86%.

Similarly, Lopatina et al. [87] used CNN along with attribution algorithms to diagnose MS patients. The network consisted of five convolutional with ReLU max-pooling layers. Once the model was built, it was trained using 132 patients’ MRI scans acquired and preprocessed with susceptibility-weighted imaging (SWI). DeepLIFT heatmaps were chosen for further investigation of the classification strategy and extract features along with LRP. The analysis revealed potential signs of MS such as veins and adjacent voxels, and common brain areas among most subjects in a class. The model achieved an accuracy of 92%.

Alijamaat et al. [88] proposed a model that combined two-dimensional discrete Haar wavelet transform (HWT) and CNN for the diagnosis of MS using MRI images. The two-dimensional discrete HWT divided the image into four sub-bands, which served as the input to the CNN networks. For parameter tuning, Adam optimizer was used. The dataset consists of 38 MS patients and 20 healthy controls obtained from Laboratory of eHealth of the University of Cyprus. The model achieved accuracy, sensitivity, and specificity of 99.05%, 99.14%, and 98.43%, respectively.

Likewise, Gaj et al. [89] developed an automated method for segmenting gadolinium-enhancing lesions from clinical MRI for MS patients. The study used two datasets: The first dataset was segmented manually, and the second was analyzed using gadolinium-enhanced lesion counts. The first dataset with manual segmentation contained 600 MRIs and was used to train and validate the model. In addition, various tests were conducted to evaluate the performance of the model such as the accuracy of lesion counts using the second dataset. Furthermore, MRI images of the gadolinium-enhancing lesions were segmented using 2D-UNet. Then, the RF classifier was used to filter these lesions. UNet models were compared using dice loss, cross-entropy loss, and bootstrapping cross-entropy loss. The model achieved accuracy of 87.7% with a 2D-UNet and RF model trained by bootstrapping cross entropy.

Ghosh et al. [90] proposed a method of diagnosing MS using four convolutional encoder networks (CENs) with various network architectures including U-Net, U-Net++, Linknet, and feature pyramid network, where all architectures had the ResNeXt-50 encoder. The dataset used contains MRI scans for 45 MS patients and was collected from two public datasets, which are the University Medical Center of Ljubljana (UMCL) and the MSSEG 2016 challenge training dataset. Preprocessing techniques were applied to the scans such as bias correction, registration, skull stripping, and visual transformation. Their findings indicated that the best MRI sequence to be used for automatic segmentation is FLAIR, since the models trained with FLAIR sequence obtained the highest dice similarity coefficients (DSCs) in the experiments, as opposed to T1 and T2 sequences. The U-Net with ResNeXt-50 model achieved the highest average DSC of 0.6678.

Al Jannat [91] developed a neural network-based system to accurately detect white matter MS lesions. The dataset contained 3766 slices of MR images from 30 patients with MS and 100 slices of healthy brain MRIs. The VGG16 model was used. Furthermore, healthy MRI scans were taken into account to obtain a more accurate result. In addition, transfer learning was used and softmax was selected as an activation function for the classification of disease progression. By utilizing FLAIR MRI scans, the system was able to optimize its total execution time. The system achieved 98.24% accuracy rate. 

To investigate using heatmap-generating methods with CNNs, Zhang et al. [92] developed a CNN model to classify subjects into three types, namely, RRMS, SPMS and healthy controls using MRI scans. The dataset included 135*3 T1-weighted, T2-weighted, and FLAIR MRI images and was acquired from 19 MS patients and 19 healthy controls. MRI slices at the start and end were excluded to improve the efficiency. Preprocessing included brain extraction, co-registration, image non-uniformity correction, and signal intensity normalization to the range 0–1. Data augmentation was also applied. The authors built six models based on ResNet50, VGG16, and VGG19. The developed models were composed of different combinations of ImageNet weights vs. random weights and used a global average pooling layer vs. fully connected layers preceding the output. Then they investigated three heatmap-generating methods, class activation mapping (CAM), gradient (Grad)-CAM, and Grad-CAM++. The training, validation, and testing split were 65%, 15%, and 20%, respectively. The VGG19 model with global average pooling and ImageNet weights achieved the highest accuracy of 95.42% and a loss value of 0.12.

Marzullo et al. [93] proposed a network-based approach for classifying MS patients into four clinical profiles. Using their structural connectivity information, which was acquired using diffusion tensor imaging and finally demonstrated as a graph, the model performance was evaluated through unweighted and weighted connectivity matrices. Specifically, 90 MS patients and 24 healthy subjects from the OFSEP consortium were studied. The study concluded that local graph metrics did not enhance the model performance, therefore implying that latent features obtained by ANN in earlier layers contain more important information. In addition, the investigators observed that graph weights representation of brain connections have paramount information to differentiate among clinical forms. The developed model achieved an F-Measure, precision, and recall of 92% (±0.01).

Ye et al. [94] proposed a method to test the hypothesis that profiles of multiple diffusion basis spectrum imaging (DBSI) metrics can distinguish lesion-defining patterns using DNN and DBSI. For the study, 38 MS patients were scanned with magnetization transfer imaging, standard conventional MRI sequences (cMRI), and diffusion-weighted imaging. Moreover, diffusion tensor imaging (DTI), magnetization transfer ratio (MTR), and DBSI were all applied to imaging voxels obtained from the regions of interest (ROIs). The developed DBSI-DNN classifier achieved accuracy of 93.4%.

La Rosa et al. [95] developed a method for detecting MS cortical lesions with 7 T MRI using a novel U-net-based deep learning technique. Two 7 T datasets were studied, the 1st consisting of 60 MS patients and the second of 20 patients. The classifier performance was tested using 0.7 mm MP2RAGE images after it was trained with 0.5 mm MP2RAGE×4, 0.7 mm MP2RAGE, or an alternation of the two. Moreover, the model generalization ability was assessed on the second external dataset and then was compared with a new method based on partial volume estimation and topological constraints (MSLAST). The model reached a true positive rate of 74% and a false positive rate of 30% for cortical lesions.

Shmueli et al. [96] proposed a new model based on EfficientNet5 and Y-net4. The model utilized attention layers to enhance performance, avoid the risk of overfitting, and extract lesion locations. Moreover, the authors used a new algorithm that is responsible for creating artificial MS lesions on healthy scans using MESE scans to increase data variability. The study was conducted on two datasets, the first consisting of nine subjects from the Lab for Advanced MRI at Tel Aviv University. The second dataset contained 30 subjects from the University Hospital of Lublijana. The model achieved accuracy of 91%.

Wang et al. [97] introduced a DenseNet-based method for MS classification. The MRIs dataset used was collected from 38 MS patients obtained from the Laboratory of eHealth of the University of Cyprus and 26 healthy controls obtained from a private source. In this study, a comparison was made between DenseNet-121, DenseNet-169, and DenseNet-201 neural networks. A composite learning factor (CLF) was also utilized that gave different learning factors to three different layers: early frozen layers, middle layers, and late replaced layers. In order to determine how layers should be allocated into the three layers, a comparison was made between four transfer learning settings (A, B, C, and D). DenseNet-201-D showed the highest result with sensitivity of 98.27± 0.58%, specificity of 98.35± 0.69%, and accuracy of 98.31± 0.53%.

Zhou and Shen [98] developed a new method of detecting multiple sclerosis lesions in MRI images using the grey-level co-occurrence matrix (GLCM) feature extraction and biogeography-based optimization (BBO) training algorithms. There were two sources of images used in this study. The first set of images came from the open access eHealth laboratory of 38 patients. Second, 681 slices from 26 healthy controls were selected. Overall, 676 MS slices and 681 HC slices were selected. As a classifier, a multilayered feedforward neural network was employed. Then, the BBO algorithm was chose to train the classifier. In addition, a 10-fold cross validation to validate the method. In general, the method demonstrated 92.75 ± 1.31% sensitivity, 92.76 ± 1.65% specificity, and 92.75 ± 1.43% accuracy. 

Following the same approach of detecting MS progression, Yoo et al. [99] explored the possibility of deriving potential features from segmented lesion masks from baseline MRI. DL techniques were used to predict short-term MS activity in patients who exhibited early symptoms more precisely than lesion volume. For this study, a dataset with 140 patients records was used. Furthermore, parameter tuning methods were used such as Euclidean distance transform and unsupervised pretraining. For feature selection and extraction, Gaussian pre-filtering, and t-distributed stochastic neighbor embedding (t-SNE) were implemented. In addition, they explored the effect of applying a 3D convolutional deep belief network (DBN) for pretraining to set the CNN model. The DBN was set using a reliable technique that takes into consideration the rectified non-linearity. The model accomplished 72.90% accuracy, 78.6% sensitivity, and 65.1% specificity.

Some studies study used OCT data for MS diagnosis. Garcia-Martin et al. [100] aimed to develop an ANN to detect MS using RNFL thickness features obtained through an OCT device. In this study, 106 MS patients and 115 healthy subjects were enrolled. The OCT device was used to acquire the RNFL thickness measures obtained from 24 equally distributed locations around the peripapillary RNFL in both eyes for each individual. The most significant locations with higher normalized importance were 315° to 330° and 120° to 135°. One eye from each subject was randomly chosen for further analysis, and 10-fold cross-validation resampling was used. The ANN successfully identified MS patients with higher accuracy than any single OCT parameter alone. The ANN achieved an AUC of 0.945. However, only good-quality scans were selected for the study, which is not always possible in clinical settings.

One study utilized retina features for the diagnosis of MS. López-Dorado et al. [101] applied CNNs to the automatic diagnosis of MS in its early stages by analyzing images obtained using SS-OCT. The study used SS-OCT images taken from 48 MS patients and 48 control subjects. Images are comprised of the following structures: complete retina, choroid, retinal nerve fiber layer, and two ganglion cell layers (GCL+, GCL++). The Cohen distance is applied to detect the structures and the regions within them that have the greatest discriminant capacity. In order to improve the training set, a deep convolutional generative adversarial network is added to the original database of OCT images. The greatest discriminant capacity is GCL++ (44.99% of image points), complete retina (26.71%) and GCL+ (22.93%). The CNN model achieved 100% accuracy, sensitivity, and specificity.

While some studies used a combination of data types for their diagnosis, Montolío et al. [102] used clinical data and RNFL thickness to build two predictive models for the diagnosis of MS and the prediction of the long-term course of disability in MS patients. The models’ input included clinical data and RNFL thickness, which was measured using OCT. They utilized various ML algorithms such as SVM, KNN, DT, MLR, NB, LSTM, and Ensemble Classifier (EC). Hyperparameter optimization was applied for each model to identify the optimal hyperparameters. For the diagnosis model, 104 healthy subjects and 108 MS patients were enrolled, where nine features were extracted from 212 subjects. The inputs to the model included clinical data and OCT parameters. Using one-hot encoding, the categorical features were encoded into numerical values. The EC achieved the highest results with an accuracy of 87.7%, sensitivity of 87.0%, specificity of 88.5%, precision of 88.7%, and AUC of 0.8775. As for the MS disability course prediction model, the model classified the subjects into two classes, worsening and non-worsening. A 10-year study was carried out for 82 MS patients. This model used data acquired at three consecutive annual visits and was intended to predict the disability course of MS patients eight years later. The inputs to the course prediction model included OCT parameters, general parameters, and MS parameters. The LSTM achieved the highest accuracy of 81.7%.

Yoo et al. [103] determined whether the CNN’s prediction accuracy can be improved by combining user-defined radiological features, such as brain volume and clinical measurements, such as EDSS. For the dataset, 140 subjects were analyzed. High image dimensionality, downsampling, unsupervised pretraining, and regularization were combined to reduce overfitting during training. In addition, it has been shown that Euclidean distance transformation and unsupervised pretraining are essential steps to effective optimization when combined with data augmentation and regularization methods. As a result, the CNN with user-defined measurements performed the best in terms of accuracy of 75.0% and AUC of 74.6%. Sensitivity and specificity were 78.7% and 70.4%, respectively. 

Vatian et al. [104] used a combination of MRI scans and clinical data to diagnose MS. They focused on fusing information acquired from a collection of MRI scans and clinical data from medical reports corresponding to these images collected from 19 patients. Accordingly, they tested the model’s performance based on early fusion, late fusion, and no fusion. They proposed an end-to-end neural network algorithm made up of two types of network architectures, namely, CNN and RCNN. The model obtained the best results when using the early information fusion with accuracy of 87.5%.

Rakíc et al. [105] aimed to develop an approach where two pipelines are utilized to classify MS lesions using MRI scans. This combined approach consisted of an unsupervised ML technique and a DL attention-gate 3D U-net network. The dataset used contained pre-contrast T1 and FLAIR brain scans from 159 MS patients and was obtained from multiple centers and through different scanners. The combined approach, which combined the outputs of the software icobrain ms 5.0 and the attention-gate U-net network, achieved better classification, detection, and segmentation of MS lesions in MRI scans than either method when used alone, especially of small juxtacortical and infratentorial lesions. The combined approach achieved the highest mean lesion-wise dice score (LWDS) of 0.64. Table 2 contains the summary of the MS diagnosis studies using deep learning. Furthermore, the table contains the testing results achieved by the proposed models. 

Karaca, Cattani, and Moonis [106] aimed to compare SVM kernels with deep learning techniques for classifying an MS dataset. This study used MR imaging data from 120 MS patients collected over the course of 3 years. The dataset consisted of MRI scans from the MS subgroups RRMS, SPMS, and PPMS. The DL and SVM kernels were used to classify the MS subgroups. In comparison with the multiclass SVM method’s kernel types, the deep learning approach had higher accuracy of 99.78% for identifying MS subgroups. 

Some studies in Section 2 utilized public datasets for their experiments. These studies along with the public datasets are shown in Table 3. Moreover, several studies in Section 2 published their codes online and are shown in Table 4.

## 3. Discussion

In this study, we reviewed studies related to the diagnosis of MS using ML and DL that were performed in the last decade. We aimed to identify the techniques and data types that have been widely used in the automated diagnosis of MS and also identified the techniques that produced significant results. Furthermore, we enlisted the open source datasets available for the MS diagnosis in Table 3. Some of the studies have also shared their source code and are mentioned in Table 4. In the section below, we first discuss the data modalities used for the diagnosis, the discussion about the studies that achieved 100% results, widely used algorithms in the literature, followed by the challenges and opportunities. 

**Figure 1:**
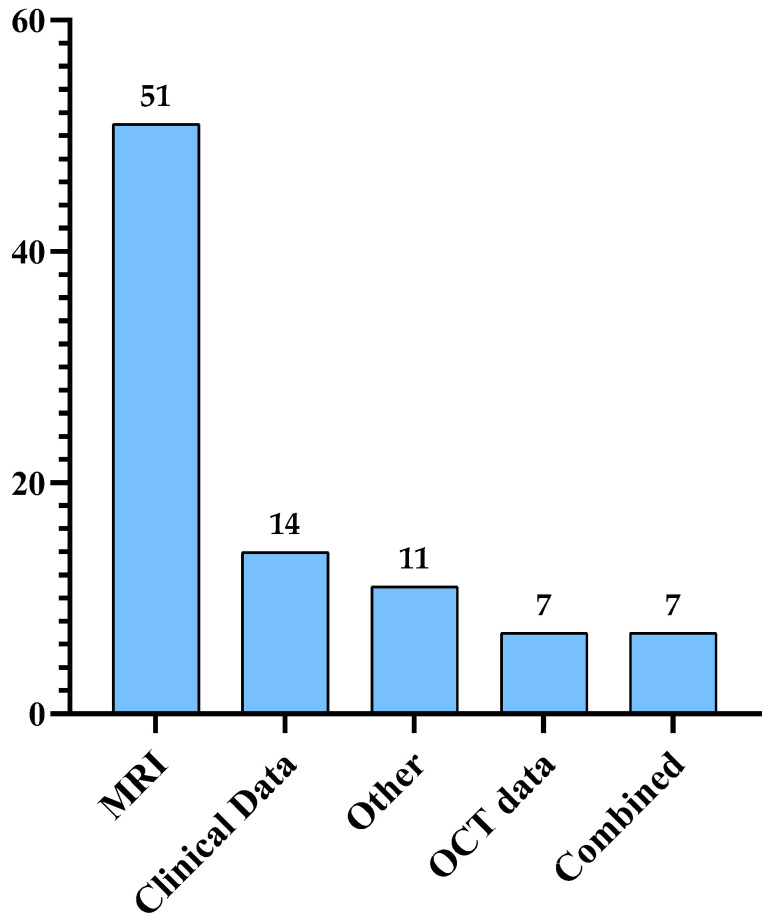
Distribution of the previous studies based on the data modalities used for the MS diagnosis.

It was found from the reviewed studies that the diagnosis of MS was performed using multiple data sources such as questionnaire data, clinical data, MRI scans, OCT data, serological measures, blood biomarkers and MEP. Some studies performed MS diagnosis using only one type of data, while others used a combination of features like clinical data, MRI, and MEP [57]. As seen in Figure 1, the highest number of studies used MRI data for the diagnosis followed by clinical data. The other common category includes the data related to RAN, MRS, MEP, brain connectivity features, EEG signals, ERG and blood biomarkers. However, the combined category contains the combination of clinical data with the other data like MRI, MEP and OCT. It can be seen from Table 1 and Table 2 that eight studies produced results of 100% for at least one measure. MRI is one of the most widely used diagnosis methods for neurological diseases because it generates accurate and fast results, and it is a secure and non-invasive procedure [107]. However, it is worth mentioning that among the studies that produced 100% results, 5 of the studies used MRI, while the other studies used different datatypes like OCT, ERG, and clinical features. Vatian et al. [104] used MRI and the radiologist notes to train the model. That study combined text mining with image analysis. Table 5 contains the details of the clinical data category used in the studies discussed in Section 2.1 and 2.2 The data consist of symptoms, demographic data such as age, weight, gender, BMI, race etc., micro-RNA structure data, medication, expanded disability status scale (EDSS), relapses, blood plasma results, lip serum, clinical history, cytokine biomarkers, and PBMC transcriptomics profiles etc. However, some studies combined different modalities like MRI and demographic data, MRI and textual information provided by the radiologist, OCT and EDSS, MRI and EDSS and demographic data. 

However, it should be noted that the studies in the literature that achieved 100% results such as [19,39,44,51,66,81,91,101] suffer from several limitations. 

Ekşi et al. [39] developed an ANN model to differentiate between low-grade brain tumors and MS lesions. The study excluded brain tumors such as oligoastrocytoma and gliomatosis cerebri that have high association with MS [108]. Furthermore, the sample size of the dataset is small. Sarbaz et al. [17] performed diagnosis using videos collected from participants while walking and used the infrared marker on their forehead to monitor their balance. The study achieved significant results but might suffer from overfitting due to the small dataset. Dorado et al. conducted two studies for the diagnosis of MS. In the first study [66], they used multifocal ERG data for the diagnosis using a sample of 21 patients. In addition to the small dataset, the samples were skewed toward MS. In the second study, Dorado et al. [101] used OCT data for analyzing the retinal changes for the diagnosis of MS. A sample of 96 patients was used to train the CNN model. Data augmentation was performed as the CNN model requires a huge dataset to adequately train the model. Despite the significant results achieved with the proposed CNN model, data augmentation sometimes leads to model overfitting. Both studies achieved specificity of 100% but suffer from using a small dataset and excluding all patient samples with other ocular diseases. Similarly, Azarmi et al. [51] achieved specificity of 100% but the number of patients in the study was 20 individuals from a hospital in Iran. The study used the patients’ fMRI data and used an SVM model for classification. 

Furthermore, Soltani et al. [80] achieved significant results with accuracy, specificity, and sensitivity above 99%, using a CNN model. The study was performed on a 72-patient sample. In addition to the significant results, the study also contains the benefit that the proposed model can also work well with blurred MRI scans. Similar results were achieved by Alijamaat et al. [88] using a dataset of 58 patients. However, they performed some preprocessing using HWT. Both previously mentioned studies utilized MRI scans from the eHealth lab dataset and used a DL model, but although the models produced considerable results but due to the small size of the dataset, the models are not robust. 

Compared with the other studies that produced 100% results, Lötsch et al. [19] used the largest dataset of 403 patients. However, the study used an invasive method for the diagnosis, and the authors needed to focus on the biomarkers that can be used for early diagnosis of MS. Merzoug et al. [44] achieved sensitivity of 100% and accuracy of 99.8% using SVM and AIS techniques. However, the main limitation of the study is that it did not contain any information about the dataset size or the distribution of MRI scan per category.

Similarly, in most of the previous studies that used MRI for the diagnosis of MS, the proposed models classified the patient sample with MS versus healthy controls [49,51,52,55,82,83,84,85,91,97,98], and the discrimination between these two classes is relatively simple. However, there is a need to devise a model that discriminates among MS and other diseases that are similar on MRI scan like brain tumors. Both diseases contain white matter in brain MRI, and this similarity sometimes might lead to the wrong diagnosis by physicians. Therefore, a model that can discriminate among these highly similar diseases will help physicians in their diagnosis. In the literature, a study performed by Siar and Teshnehlab [81] proposed a CNN model that discriminate among the two tumors and MS. The study achieved significant results, but the limitation of the study was that the dataset was not large. Additionally, Casino et al. [74] proposed a model that could discriminate between MS and ADHA. The diseases share similarities, and therefore, it is significant to develop a model that can discriminate between them. Another significance of this study was that most of the previous studies focused on the adult patient sample, whereas these authors focused on children using the mRNA expression data. 

Macin et al. [52] achieved very high sensitivity but the study used manual feature extraction. Moreover, a KNN model was used, which is a lazy learner that requires high testing time and high space. Similarly, Deshpande et al. [55] also achieved the high sensitivity but PCA feature extraction has been used that can’t handle the nonlinear data. Furthermore, Acar et al. [85] used a very small dataset, and their model could not be generalized. Additionally, Ye et al. [94] suffers from imbalance along with the small dataset. 

Wang and Lima [82] used multiple augmentation techniques to better train the model. However, due to extensive augmentation, the model might have suffered from overfitting; augmentation techniques generate synthetic data. Shmueli et al. [96] also utilized data augmentation with many fewer patients; in addition, the study used a single center data, while Rosa et al. [95] utilized multicenter data. However, that study performed manual segmentation. 

Age is identified as one of the significant factors for the diagnosis of MS because age brings changes in the brain [91]. The studies that merely used MRI did not consider this factor. Therefore, there is a need to integrate different data modalities such as MRI, OCT, clinical, and textual. 

In addition to the diagnosis, there are some studies that perform prognosis or discriminate among the different types of MS such as RRMS, PPMS, and SPMS. Cattani et al. [106] achieved accuracy of 99.78 for classifying different types of MS, but that study suffers from huge imbalance. Zurita et al. [54] proposed a classification model for RRMS patients. The performance of the model was not significant for patients with different levels of disability. In term of ML algorithms used for MS diagnosis, SVM is the most widely used, followed by RF. However, the best-performing algorithm is RF. As for DL algorithms, the most frequently used algorithm with the best performance is CNN.

**Figure 2:**
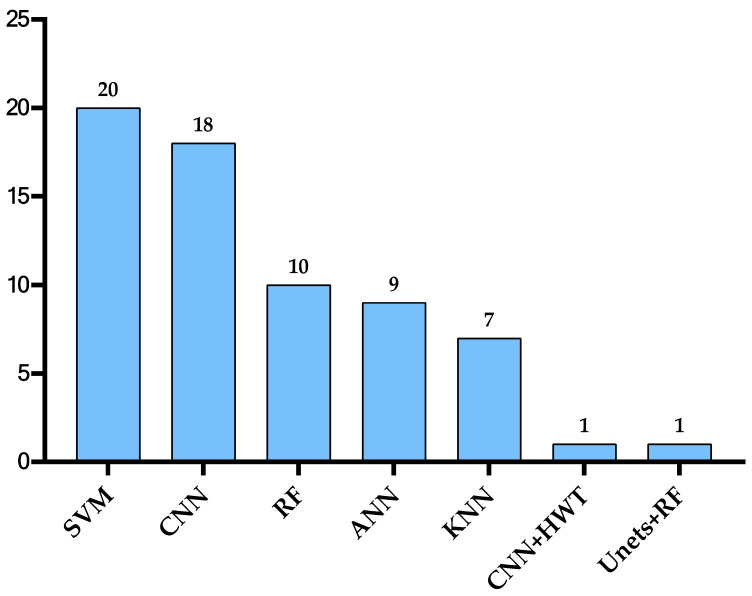
Widely used ML and DL methods in the previous studies.

**Figure 3:**
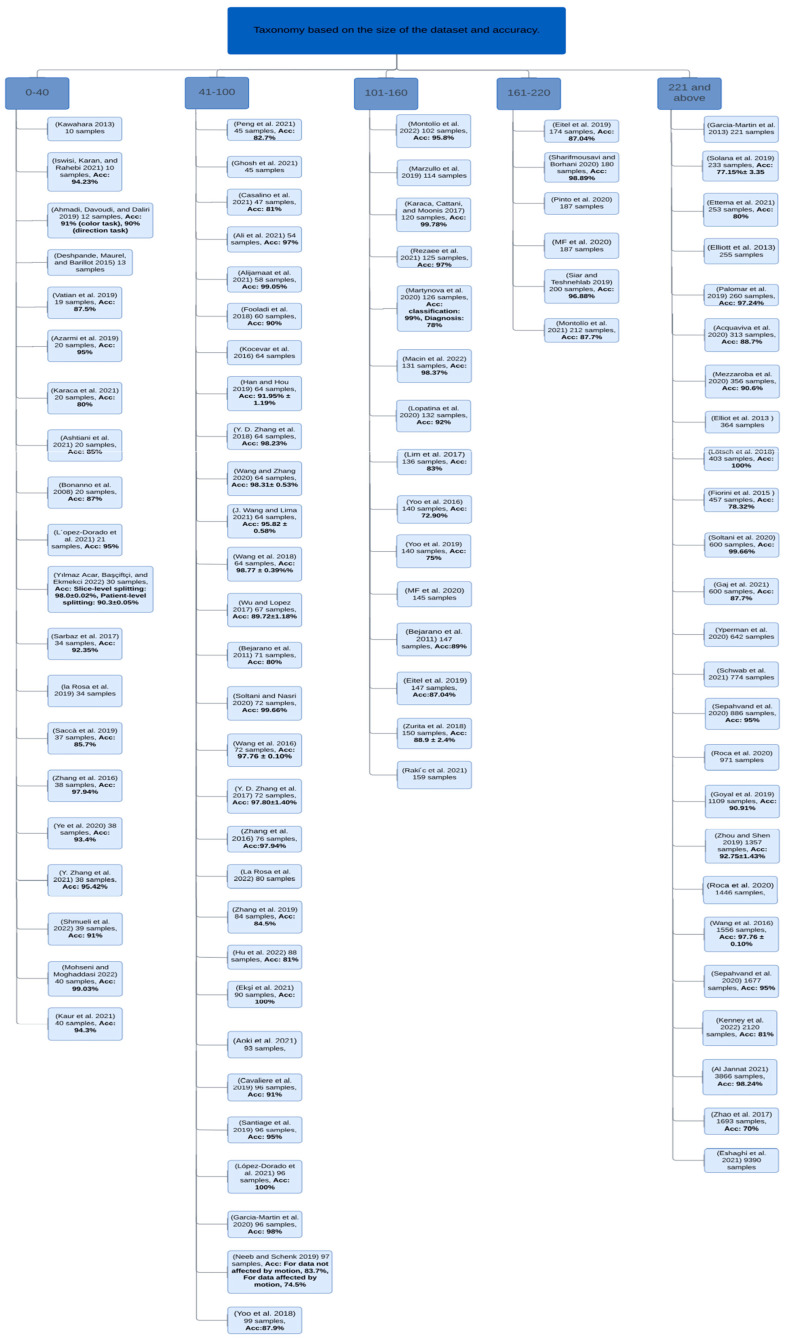
Taxonomy of studies based on dataset and accuracy.

Moreover, most of the studies utilized datasets that consisted of MRI scans, although several studies depended on clinical data to diagnose the disease. The used dataset sizes ranged from 10 to 9390 instances. However, some of the studies did not mention the size of the dataset they used. Figure 2 contains a summary of the widely used ML and DL techniques in the previous studies. Figure 3 contains the taxonomy of the related studies using dataset size and accuracy (four studies did not specify the number of patients, and therefore, those studies are not included in the figure). The largest number of studies have datasets in the range of 41–100 or 221 and above. Furthermore, most of the studies with the dataset size 41–100 produce significant results. 

### 3.1. Challenges

#### 3.1.1. Identifying the Disease

MS is not a disease that can be identified easily as there are no tests, symptoms, or physical findings that can be used to accurately diagnose it. Multiple methods are used to support the diagnosis process including MRI scans, analyzing the patient’s medical history, blood tests, and spinal fluid analysis [12]. However, these methods are tedious, time-consuming, and prone to errors. There are, however, implications for AI in the disease diagnosis: specifically, the DL and ML models are promising techniques for accurately identifying MS [12,109]. These tools can be used to assist clinicians in their diagnosis. 

#### 3.1.2. Privacy and Confidentiality of the Patients’ Data

The sensitivity of the collected patients’ data raises several privacy and confidentiality concerns, as acquiring the data needed to build the models while protecting patients’ privacy is difficult. In addition, the patient’s identity may be susceptible to being revealed through the information accompanying the MR imaging data. In brain imaging, structural images may allow for the reconstruction of faces, thus exposing the patient’s identity. To solve these issues, face removal and scrambling can be employed. However, these techniques may affect the succeeding image analysis. Consequently, protecting patients’ privacy while collecting their information continues to be a major challenge that needs to be addressed appropriately [110].

#### 3.1.3. Reliability of the Models

AI-based diagnosis systems may suffer from a certain degree of error and bias [111]. As a result, these models cannot be blindly trusted with their diagnosis results. This may stem from ill-trained models resulting from multiple factors including noisy data, unbalanced datasets, and biased data. 

#### 3.1.4. Issues in Collected Data: Size, Noise, Imbalance

In order to develop an automated MS diagnosis model, a large dataset is required to ensure the reliability of the developed model. However, obtaining a large dataset is not a simple task as evidenced by the small datasets used in most of the papers in Section 2. The difficulty of obtaining a large dataset stem from issues in finding participants suffering from MS and the amount of time it takes to collect the necessary data from each of them. Moreover, it is important to consider the possible differences between data collected for a study and data collected in real-world contexts, since real-world data tend to have some degree of contamination like missing values and measurement errors that are left untreated. This might limit the use of such models in real clinical settings [109]. In addition, the same patient may follow up with more than one clinician from different hospitals. Hence, the longitudinal follow up of the patients is lacking and eventually, important set of chronological data will be lost too.

#### 3.1.5. Model Interpretation

Despite all efforts, it is still impossible to understand and explain neural network decisions. Future studies are required for explaining how DL algorithms perform their predictions. Scientists may also be able to discover and understand new pathophysiologic knowledge from AI models. Therefore, researchers are encouraged to interpret and explain the inferencing of their developed ML models. Kim [112] argues that transparent ML models can earn the trust of their users and thus encourage the adoption of autonomous systems in clinical settings. 

### 3.2. Opportunities

#### 3.2.1. More Secure Platforms

It is crucial to implement security solutions and policies that will help ensure the confidentiality and reliability of health care systems that collect patients’ data. Since these data may be private, it is important to protect it against data leakage.

#### 3.2.2. New, Better Algorithms

There are relatively few studies regarding the use of AI-based techniques for MS diagnosis. This makes it a promising area for future research, where researchers can experiment with various algorithms to build models with higher performance. Moreover, the combination of CNN with other DL algorithms can be explored [80].

#### 3.2.3. Prognosis

Machine learning is capable of predicting MS disease course on an individual level [109]. Numerous methods have been introduced in the field of MS prognosis. Nevertheless, no model succeeded in entering routine practice. The users of these models, such as neurologists, need to be more comfortable using them. Moreover, no study has developed models predicting the course of MS with performance reliable to use in clinics. Therefore, further research is encouraged in this area to reach the goal of clinically usable and reliable automated systems that predict the individual natural course of MS disease [15], especially the scarcely studied cognitive prognosis [109]. In addition to predicting the natural course of the disease, the simulation of treatment response can also be implemented to predict how the natural course of MS changes after taking disease modifying therapy [109].

#### 3.2.4. Combine Multiple Data Types for Diagnosis

Several studies recommended incorporating multiple data types for more accurate diagnosis, such as combining OCT data with MRI, EP, or CSF [102]; combining clinical data with lesion loads and metabolic features [60]; combining clinical characteristics and multimodal imaging [40]; and incorporating features including neuroimaging measures and blood and genetic biomarkers [59].

#### 3.2.5. Use of OCT Data

OCT data were only been used in a few studies and showed promising results, [61,62,63,101,102]. Palomar et al. [61] proved that RNFL thickness can be used as a biomarker for MS diagnosis since it attained precision higher than 95%. Furthermore, it is recommended to explore OCT parameters in a real clinical setting as they are usually obtained by specialized devices with good-quality scans that is not always possible in the real world [61,62].

#### 3.2.6. Using Larger and Multicenter Data

Numerous studies suffer from limited data sizes [40,66,69,70,77,87,90]. In addition, many studies had access to data from only one center [40,62,70], which may introduce bias. Therefore, the use of larger and multicenter data is encouraged as it improves the reliability of the diagnostic models. 

#### 3.2.7. Commercialization

Earlier detection and better monitoring of MS through AI has proven to result in better clinical outcomes and, subsequently, improving the health care system and quality of life of MS patients. The commercialization of the most accurate and cost-effective AI platforms along with utilizing the advances in data collection technologies will revolutionize the way clinicians deal with their patients providing a platform for precision-based medicine. 

## 4. Conclusions

This paper attempted to provide a comprehensive review of the previous contributions achieved by researchers in the automated diagnosis of multiple sclerosis. Employing AI solutions and utilizing ML algorithms in the medical field has enhanced the medical applications for MS diagnosis. In this paper, we identified several ML methods used for MS diagnosis and discovered that the most used techniques were SVM, followed by RF and CNN. Moreover, we discussed the challenges and opportunities for diagnosing MS to find areas where researchers and practitioners can improve their approaches. 

All research opportunities identified in this research can be explored in the future. However, the current authors’ perspective aims for more understanding of MS in different contexts. That is, ML algorithms will be used for the diagnosis and prognosis of the disease using real datasets. These may be demographic, clinical, and lab or machine data (radiology, patient monitoring data, etc.). Moreover, new features will be explored to identify potential predictors.

## Figures and Tables

**Table 1:** Summary of related studies using machine learning.

Ref	Method	Data Type	Dataset Size	Num of Features	Specificity	Sensitivity	F1-Score	Accuracy
[16]	OLS	Clinical data	457 subjects	91	-	-	70.1%	78.32%
[17]	ANN with a tan-sigmoid transfer function	Video recordings	34 subjects	11	82.63%	97.77%	-	92.35%
[18]	ANN	Clinical data	253 subjects	-	74%	93%	-	80%
[19]	RF	Clinical data	403 subjects	8	100%	100%	-	100%
[20]	KNN, DT, XGB, gNB, and RF	Clinical data	126 subjects	45	-	-	-	Diagnosis: 99% (CSF) ≥ 96 (Serum) Classification: 78% (Serum) 69% (CSF)
[25]	SVM	Clinical data	187 subjects	10	77%	76%	20%	-
		Clinical data	145 subjects	18	81%	84%	-	-
[26]	SVM	Cognitive task-related fMRI	20 subjects	8 (global) 6 (local)	95.83%	68.75%	-	85%
[27]	GBM	Gait data and raw data	40 subjects	21	-	88.2%	93.8%	94.3%
[21]	RF	Clinical data	54 subjects	-	96.47%	96.4%	95.6%	97%
[28]	DT	Clinical data	136 subjects	-	-	91%	-	83%
[29]	SVM	Clinical Data	356 subjects	-	-	-	-	90.6%
[30]	SVM	Raw walkway sensor data, Demographic and symptoms	88 subjects	11 features	-	81%	87%	81%
[31]	Bayesian classifier + RF	MRI	255 subjects	63	-	99%	-	-
[32]	ANN	MRI	72 subjects	5	97.82 ± 1.60%	97.78 ± 1.29%	-	97.80 ± 1.40%
[33]	BWT, RKPCA, and LR	MRI	72 subjects	-	98.25 ± 0.16%	97.12 ± 0.14%	-	97.76 ± 0.10%
[34]	SWE with KNN	MRI	76 subjects	-	99.32%	96.15%	-	97.94%
[35]	RF	MRI	84 subjects	-	50%	94%	-	84.5%
[36]	SVM, RF	MRI	37 subjects	1	66.7%	-	-	85.7%.
[37]	SVM	MRI	72 images	-	-	-	-	77.83%
[38]	ELM	MRI	125 subjects	48	-	-	-	97%
[39]	ANN	MRI	90 subjects	17	100%	100%	-	100%
[40]	SVM	MRI	45 subjects	972	84.1%	80.9%	-	82.7%
[41]	SuStaIn	MRI	9390 subjects	18	-	-	-	-
[43]	EDT	MRI	45 images	-	-	-	-	98.5%
[44]	AIS + SVM	MRI	-	-	83.8%	100%	-	99.8%
[45]	BRNN	MRI	93 subjects	-	95.2%	77.8%	-	-
[46]	Hybrid Watershed-Clustering algorithm	MRI	20 subjects	13	87%	77%	-	87%
[47]	HHO + FCM	MRI	10 subjects	-	93.34%	89.56%	-	94.23%
[48]	KNN and SVM-polynomial kernel	MRI	192 images	18	-	-	-	96.55%.
[49]	ANN	MRI	64 subjects	-	91.98% ± 1.36%	91.91% ± 1.24%	-	91.95% ± 1.19%
[50]	LR	MRI	67 subjects	-	-	-	-	89.72 ± 1.18%
[51]	SVM	fMRI	20 subjects	8 and 9	100%	87.5%	-	95%
[52]	KNN	MRI	131 subjects	768 features	99.60%	96.46%	97.89%	98.37%
[53]	Multivariate supervised ML models, KNN	MRI	97 subjects	-	-	-	-	For data not affected by motion, 83.7% For data affected by motion, 74.5%
[54]	SVM	MRI	150 subjects	30 features	89.7 ± 3.6%	88.0 ± 2.7%	-	88.9 ± 2.4%
[55]	Dictionary Learning	MRI	13 subjects	-	-	99.5%	-	-
[56]	RF	MRI	99 subjects	-	88.6%	87.3%	-	87.9%
[57]	Bayesian, RD, simple LR, and NNets	Clinical data, MRI and MEP	DS1 71 subjectsDS2 96 subjects	-	77%	92%	-	80%
[58]	SVM-RBF	Clinical data	64 subjects	-	-	-	91.8 for HC-CIS. 75.6 for CIS-RR. 70.6% for RR-PP.	-
[59]	LR, SVM	Longitudinal clinical and MRI	1693 subjects	-	68%	71%	-	70%
[60]	LDA and SVM-RBF	Clinical data with lesion loads and MR metabolic features	592 images	-	-	-	71% for CIS vs. RR. 72% for CIS vs. RR + SP. 85% for RR vs. PP. 87%for RR vs. SP.	-
[61]	DT with AB	OCT data	260 subjects	-	97.86%	95.52%	-	97.24%
[62]	SVM-Gaussian quadratic kernel	OCT data	96 subjects	3	92%	89%	-	91%
[63]	FFNN	OCT data	96 subjects	-	98%	98%	-	98%
[64]	KNN	OCT data	102 subjects	-	97.2%	94.4%	-	95.8%
[65]	SVM	OCT data	2120	-	80%	81%	-	81%
[66]	SVM	ERG	21 subjects	40	100%	93%	-	95%
[67]	SVM	EEG	40 subjects	-	99.18%	98.90%	-	99.03%
[68]	OSELM	EEG	12 subjects	-	96% (color task) 96% (direction task)	83% (color task) 82% (direction task)	-	91% (color task) 90% (direction task)
[69]	KNN	PS EEG signals	20 male subjects	20	88.9%	72.7%	-	80%
[22]	ADAboost-FT	Clinical data	313 subjects	-	77.8%	94.3%	-	88.7%
[23]	RF	Clinical data	1109 subjects	8	85.7%	75.6%	-	90.91%
[24]	SVM	Clinical data	180 subjects	-	-	98.98%	-	98.89%
[70]	KNN	mfVEP recordings	96 subjects	6	-	-	-	95%
[71]	RF	MEP	642 subjects	7700	-	-	-	-
[72]	SVM	Brain connectivity features	233 subjects	42	80.01% ± 3.77	74.27% ± 7.85	75.99% ± 4.37	77.15% ± 3.35
[73]	LR, multiple LR, and non-linear non-parametric RFs	Spinal cord features	-	13	-	-	-	-

**Table 2:** Summary of related studies using deep learning.

Ref	Method	Data Type	Dataset Size	No of Features	Specificity	Sensitivity	F1-score	Accuracy
[102]	LSTM	clinical data and OCT data	212 subjects	9	88.5%	87.0%	-	87.7%
[100]	ANN	OCT data	221 subjects	24	-	-	-	-
[75]	AAM	Smartphone-derived digital biomarkers	774 subjects	-	73%	83%	80%	-
[76]	CNN	MRI	34 subjects	-	-	-	-	-
[77]	CNN	MRI	147 subjects	-	81%	93.08%	-	87.04%
[78]	CNN	MRI	886 subjects	-	97%	69%	-	95%
[79]	CNN + RF + Mainfold learning	MRI	1446 subjects	65	-	-	-	-
[80]	CNN	MRI	72 subjects	-	99.33%	99.98%	-	99.66%
[81]	CNN	MRI	200 subjects	-	100%	94.64%	-	96.88%
[82]	6-layer CNN	MRI	64 subjects	-	95.67 ± 0.92%	95.98 ± 0.46%	95.81 ± 0.57%	95.82 ± 0.58%
[83]	14-layer CNN	MRI	64 subjects	-	98.76 ± 0.58%	98.77 ± 0.35%	-	98.77 ± 0.39%
[84]	CNN	MRI	64 subjects	-	98.24%	98.22%	-	98.23%
[85]	CNN	MRI	30 subject	200	Slice-level splitting: 98.3 ± 0.03% Patient-level splitting: 90.1 ± 0.09%	Slice-level splitting: 97.9 ± 0.03% Patient-level splitting: 90.5 ± 0.05%	-	Slice-level splitting: 98.0 ± 0.02% Patient-level splitting: 90.3 ± 0.05%
[86]	ENN-AIC	MRI	60 subjects	-	-	92%	-	90%
[87]	CNN, LRP	MRI	132 subjects	-	-	-	-	92%
[88]	HWT +CNN	MRI	58 subjects	-	98.43%	99.14%	-	99.05%
[89]	2D-UNet and RF	MRI	600 subjects	75	-	-	-	87.7%
[103]	9-layer CNN	MRI and clinical data	140 subjects	11	70.4%	78.7%	-	75.0%
[91]	VGG16	MRI	3866 subjects	-	95.45%	100%	-	98.24%
[92]	VGG19	MRI	38 subjects	-	-	-	-	95.42%
[93]	Graph based NN	MRI	114 subjects	-	-	92% (±0.01)	92% (±0.01)	-
[94]	DNN	MRI and clinical data	38 subjects	-	-	-	-	93.4%.
[95]	U-Net	MRI	80 subjects	-	-	-	-	-
[96]	EfficientNet5 + Y-net4	MRI	39 subjects	-	-	-	-	91%
[97]	DenseNet-201	MRI	64 subjects	-	98.35 ± 0.69%	98.27 ± 0.58%	98.30 ± 0.53%	98.31 ± 0.53%
[98]	FFNN	MRI	1357 subjects	-	92.76 ± 1.65%	92.75 ± 1.31%	-	92.75 ± 1.43%
[99]	CNN	MRI	140 subjects	-	65.1%	78.6%	-	72.90%
[74]	ANN	M-RNA expression Data	47 subjects	40	-	-	-	81%
[101]	CNN	OCT data	96 subjects	64	100%	100%	100%	100%
[104]	CNN and RNN	MRI and textual clinical records	19 subjects	-	-	-	-	87.5%
[105]	Combined approach	MRI	159 MS patients	-	-	-	-	-
[106]	DL	MRI	120 subjects	-	-	-	-	99.78%

**Table 3:** Publicly available datasets.

Ref.	Dataset Name
[16]	Private + eHealth Lab
[18]	Private + eHealth Lab
[19]	Private + eHealth Lab
[20]	Private + eHealth Lab
[21]	Private + eHealth Lab
[85]	LabIT
[33]	Private + eHealth Lab
[34]	Private + eHealth Lab
[43]	2008 MICCAI MS Lesion Segmentation Challenge
[44]	Brainweb
[47]	“Whole Brain Atlas” image database
[48]	Private + eHealth Lab
[49]	Private + eHealth Lab
[75]	Floodlight Open
[88]	eHealth Lab
[96]	University Medical Centre Ljubljana (UMCL)
[98]	Private + eHealth Lab

**Table 4:** Open-source codes weblinks.

Ref.	Link
[41]	https://github.com/ucl-pond/pySuStaIn (accessed on 26 July 2022). Note: they used the code at commit 54b92b154acc9d8757751edea50d1fcfab672015.
[77]	https://github.com/derEitel/explainableMS (accessed on 26 July 2022).
[89]	https://github.com/sibajigaj/Gad_lesion_segmentation (accessed on 26 July 2022).

**Table 5:** Summary of the previous studies that used clinical data.

Ref.	Clinical Data Type
[16]	Demographic, mobility, fatigue, cognitive performance, emotional status, bladder continence and quality
[19]	Demographic, EDSS, medication, lip serum
[20]	Cytokine Biomarkers
[25]	Demographic, EDSS, medication, symptoms, Clinical History
[21]	m-RNA expression data
[28]	KP metabolic
[29]	blood sample-inflammatory, oxidative, nitrosative stress, medication, demographic
[30]	raw walkway sensor data, demographic data, symptoms
[57]	EDSS, disability progression, and new relapses
[58]	EDSS + demographic
[59]	EDSS + demographic
[60]	EDSS + demographic
[22]	PBMC transcriptomics profiles
[23]	Serum Cytokines + Demo +EDSS
[24]	Plasma sample
[18]	exhaled breath analysis using an electronic nose
[102]	Age, sex, best corrected visual acuity, MS parameters
[103]	demographic + demyelinating symptoms
[94]	Demographic + MS-subtype and MS-lession type

## Data Availability

Not applicable.
